# On an Electrical Phenomenon Observed in Cholera

**Published:** 1849-01

**Authors:** J. C. Atkinson

**Affiliations:** Westminster


*On an Electrical Phenomenon observed in Cholera.* By J. C ATKINSON, Esq., M. R. C. S., &c., Westminster.

I am desirous at the present moment of directing the attention of your numerous scientific readers to a very interesting phenomenon, more or less present in the collapse stage of cholera, which seem to have hitherto escaped the observation of medical men—viz., animal electricity, or phosphoresence of the human body. My attention was attracted to the subject during the former visitation of that fearful disease in the metropolis. It was indeed singular to notice the quantity of electric fluid which continually discharged itself on the approach of any conducting body to the surface of the skin of a patient laboring under the collapse stage, more particularly if the patient had been previously enveloped in blankets; *streams of electricity* many averaging *one inch and a half* in length, could be readily educted by the knuckle of the hand when directed to any part of the body, and these appeared, in colour, effect, crackling noise, and luminous character, similar to that we are all accustomed to observe when touching a charged Leyden jar. I may remark the coincidence. that simultaneously with the heat of the body passing off, the electricity was evolved; and I am therefore led to ask question—Are not heat, electric and galvanic fluids *one* and the same thing? Does not the fact of the passing off of both imponderable agents at one and the same time strengthen this conclusion?

Again: are not the whole of what we call *vital* phenomena produced by certain modifications of the electric-galvanic-magnetic matter and motions? and do we not find that these *vital* phenomena are continuously affected by the relative state of the surrounding electric medium? To what can we attribute the present fluctuating condition of the barometer, if not to it?

We *know* what wonderful *decomposing* action galvanism had on alkalies, under the hands of the illustrious Humphrey Davy; but we do *not know*, nor have we any conception in the present state of knowledge, of the *decomposing* action of electric matter of the atmospheric air, in various conditions, on the fluids generally of the animal body. Chemistry has failed in pointing out any ponderable material as the exciting cause of epidemic diseases,

In the treatment of Cholera, all are agreed that *non-conducting* substances on the surface of the skin aid essentially the cure; and during the disturbed state of the atmosphere, for the purpose of retaining the electricity continually eliminating in the system, we are told to wear woollen bandages, flannel, and gutta percha soles, so as to insulate as much as possible the body, to prevent heat---the electric fluid---from passing off.—*London Lancet* in Med. Exam.

---

